# Social Skills in Children at Home and in Preschool

**DOI:** 10.3390/bs9070074

**Published:** 2019-07-08

**Authors:** Maryam Maleki, Abbas Mardani, Minoo Mitra Chehrzad, Mostafa Dianatinasab, Mojtaba Vaismoradi

**Affiliations:** 1 School of Nursing and Midwifery, Shahroud University of Medical Sciences, Shahroud 3614773955, Iran; 2 Department of Pediatric Nursing, School of Nursing and Midwifery, Guilan University of Medical Sciences, Rasht 4199613776, Iran; 3 Department of Epidemiology, Center for Health Related Social and Behavioral Sciences Research, Shahroud University of Medical Sciences, Shahroud 3614773955, Iran; 4 Faculty of Nursing and Health Sciences, Nord University, Bodø 8049, Norway

**Keywords:** social skills, preschool children, parent, teacher, parent–teacher agreement

## Abstract

Preschool age is a crucial period for social development. Social skills acquired during this period are the basis for future life’s success. This study aimed to investigate the level of social skills in preschool children at home and in preschool and to examine the association between children’s social skills and environmental and cultural backgrounds. A cross-sectional study using a multistage cluster sampling method was conducted on 546 children studying in the preschool centers of an urban area of Iran. Data were collected through demographic and social skill questionnaires from parents and teachers. Our findings showed that the social skills of girls were more than those of boys at home. Further, the majority of children had a moderate level of social skills from the parents’ and teachers’ perspectives. There was a modest parent–teacher agreement in most domains of social skills. Moreover, a statistically significant association was reported between children’s social skill domains and the child’s birth rank, father’s age, father’s job, teacher’s age, teacher’s education, teacher’s experience, and preschool classroom in terms of the numbers of children and the type of classroom. Accordingly, the risk of problems with social skills was reported to be relatively low. Therefore, more attention should be given to the family status and the teacher’s and preschool center’s characteristics to improve social skills in children.

## 1. Introduction

Healthy children create the future of any society [1]. Therefore, there is a need to pay attention to their health development process and mental health [2]. Acquiring social skills is a fundamental part of mental health [3]. Social skills are learned behaviors based on social rules and enable individuals to interact appropriately with others in society [4]. Further, based on another definition, social skills are defined as a component of social competence and a general measure of the quality of social behavior [5]. Social skills enable human beings to develop social relationships in various life stages [6].

Social skills enable social adaptation, create and maintain existing social relationships, and have long- and short-term effects over an individual’s life [7]. Therefore, preschool age is a crucial period for the development of social skills among children [8]. Therefore, the development of social skills enables children to create successful relationships with others, helps with school readiness, and improves adaptation to the formal school setting as well as academic performance [4,9]. A lack of social skills in children leads to feelings of loneliness, subsequent mental and behavior problems, poor interactions with their parents, teachers, and peers, and school maladjustment [10,11,12]. Therefore, it is required to investigate preschool children’s social skills and identify social deficiencies to design interventions aiming at the improvement of their social skills and quality of life, and adaptation to the environment at early ages. The assessment of social skills is performed through the measurement of cooperation, assertion, and self-control [13].

Preschool and home are important life settings that play an essential role in the development of children’s social abilities [14], and parents and teachers have important roles [15]. The development of social skills initially is started at home at interpersonal levels through interactions with parents [15]. Next, children enter preschool as the first social environment and continue the process of socialization [2,16]. Since children spend most of their time with teachers during preschool [17,18], teachers perform caregiving functions similar to those of parents in terms of preserving their safety, relieving their stress, and educating them in cases of misbehaviors [19]. Children practice social skills within teacher–child interactions [20] and use them in subsequent interactions at home with parents [21]. Similarly, children apply social skills acquired at home in subsequent interactions with teachers and peers at school [20]. Accordingly, teachers and parents are considered influential forces in the development of children’s life skills [22,23] and are in the best position for the provision of a reliable evaluation of children’s social skills [24]. Teachers interact with children in different various situations, in which various social skills are needed [17,24]. Teachers are able to observe a variety of social behaviors in children, which parents usually lack the required experiences to perform. On the other hand, parents’ knowledge of children’s behaviors goes beyond the classroom setting [25]. Therefore, a comprehensive evaluation of children’s social skills needs the assessment and comparison of both parents’ and teachers’ perspectives [18]. In addition to parents’ and teachers’ characteristics, the family socioeconomic status, home, and school environment influence the evaluation of social skills in children [11,16,26].

The development of social skills starts at an early age and is different between males and females [22]. For example, in girls, it is faster than boys [5,27,28], but Iranian studies have found no relationships between gender differences and the development of social skills [29,30]. In addition, the cultural background influences the display of social skills in different social environments [31]. The socialization process in non-Western contexts happens through adherence to the expectations of parents and the society, but in industrialized societies, authoritarian status has a lower effect on the parent–child relationship [32]. Nevertheless, a few studies have examined social skills in Iran. Therefore, there is a need to assess gender differences in relation to social skills in various cultural backgrounds. 

In the preschool period, 3–6-years-old Iranian children enter kindergartens and preschool centers [33]. This one-year period is optional, and children at the age of six years would be able to enter the elementary school [34]. Despite the growing importance of social skills of preschool children, little is known around social skills in different settings and the association between social skills and cultural backgrounds in the Iranian context. Therefore, this study was conducted to improve our understandings of social skills in preschool children, identify differences between girls and boys in terms of social skills, find the parent–teacher agreement of social skills in preschool children at home and in preschool, and examine the association between social skills and the environmental and cultural backgrounds in the Iranian context.

## 2. Methods

### 2.1. Design and Participants

This cross-sectional study was conducted on preschool children at preschool centers in an urban area of Iran from 2015 to 2016. They were selected based on the inclusion criteria of an age of six years (born from 23 September 2009 to 23 September 2010), living in the city of Rasht, acquaintance between the child with the teacher for at least 3 months, and the preschool center being under the supervision of Rasht Ministry of Education (regions 1 and 2) and the Welfare Organization. Known physical and mental health problems, and living with one parent or another caregiver were considered exclusion criteria.

The results of the study by Sheikhzakaryaie et al. on children at preschools in the city of Tehran were used to estimate the sample size [30]. Other variables for this estimation were the standard deviation of 19 for the social skill scales of preschool children, 95% confidence level, and assuming that the accuracy of estimating the mean score of children’s social skills was at least 2. Therefore, using a statistical formula and the cluster model design (design effect = 1.5), the sample size was estimated to be 525 people as follows:

$$n=\frac{z^{2}s^{2}}{d^{2}}\Rightarrow n=\frac{1.96^{2}\times 19^{2}}{2^{2}}=350\Rightarrow n=350\times 1.5=525.$$

**Figure 1:**
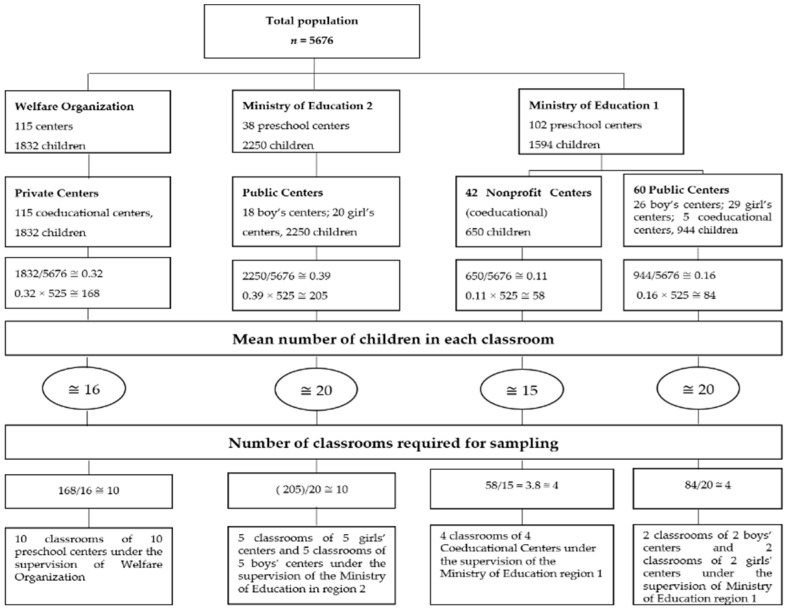
Process of sampling in this study.

For two-stage random cluster sampling, data regarding the number of preschool age children at each preschool center were collected. The number of preschool age children was classified on the basis of preschool centers supervised by the Ministry of Education regions 1, 2, and the Welfare Organization. They consisted of boys’, girls’, and coeducational preschool centers of the public, both private and nonprofit type. The number of children in each preschool center was divided into their total population and multiplied by the sample size (n = 525). Further, the number of children from public, private, and nonprofit preschool centers was calculated, and then it was divided into the average number of children in each classroom to calculate the number of classes (n = 28). The preschool centers were selected using a random method, and from each center, one classroom was chosen randomly to recruit the samples (Figure 1).

### 2.2. Measurement

The demographic data and the social skills rating system (SSRS) questionnaires were used for data collection. 

#### 2.2.1. Sociodemographic Data Questionnaire

Three questionnaires were designed by the researchers through a review of literature to examine the sociodemographic variables of children. The validity of these questionnaires was confirmed using face and content validity methods. They included the child’s demographic questionnaire consisting of questions about the child’s gender, birth rank, number of children in the family, going to the kindergarten, and presence of physical and mental health issues. Further, the child’s family questionnaire included questions about parents living with a spouse, parent’s age, parent’s education level, parent’s job, and family income. The teacher’s demographic questionnaire included questions about the teacher’s age, education level, and teaching experience, number of children in each classroom, type of classroom (single-sex, coeducational), and characteristics of the preschool center type.

#### 2.2.2. Social Skills Questionnaire

The social skills rating system (SSRS) was used to investigate social skills in preschool children from the parents’ and teachers’ perspectives. The SSRS is a comprehensive and psychometrically tested tool for evaluating social skills among preschool children [35]. The social skills rating system–teacher (SSRS–T) was evaluated in terms of internal consistency (r = 0.93–0.94). The test–retest coefficient within the four-week interval was reported to be 0.85. For the social skills rating system–parent (SSRS–P), internal consistency was reported 0.87–0.90, and the reliability coefficient of test–retest within a four-week interval was 0.87 [13]. Shahim in Iran reported the internal consistency of the SSRS–T and SSRS–P to be 0.71 and 0.88, respectively. In addition, the test–retest reliability coefficients within a 4–5-week interval for the SSRS–T and the SSRS–P were 0.71 and 0.70, respectively [36]. 

The SSRS is a 3-point Likert scale (0 = Never, 1 = Sometimes, and 2 = Very often). The SSRS–T is a standardized 30-item questionnaire consisting of three subscales of ‘cooperation’, ‘assertion’, and ‘self-control’. The cooperation domain includes 19 questions with a score range from 0 to 38, the assertion domain includes 8 questions with a score range between 0 and 16, and the self-control domain includes 3 questions with a score range from 0 to 6. Furthermore, the total score of social skills in the SSRS–T is calculated through summing up all subscale scores with a range of scores from 0 to 60. The SSRS–P is a standardized 39-item question with three subscales of ‘cooperation’ (19 questions with a score range from 0 to 38), ‘assertion’ (16 questions with a score range from 0 to 32), and ‘self-control’ (4 questions with a score range from 0 to 8). Further, the total social skill score of the SSRS–P is calculated through summing up all subscale scores with a range of scores from 0 to 78 [37,38]. For both the parent’s and teacher’s forms, the SSRS manual provides cutoff points to categorize children scores in each subscale and the total social skill score into three categories of low, moderate, or high. Scores within one standard deviation of the mean indicate the moderate level. Scores with one standard deviation below or above the mean category fall into the low or high levels, respectively [13,39]. 

### 2.3. Data Collection

Out of 255 preschool centers, 28 preschool centers were randomly selected (Figure 1), and from each center, only one classroom was chosen randomly. Data were collected within the school from December 2015 to January of 2016. The children’s names were coded, and the teachers were asked to fill out the teachers’ demographic and the SSRS–T questionnaires. In addition, the children’s parents were requested to complete the child’s family and SSRS–P questionnaires during their referral to the preschool centers. Given the probability of sample’s attrition, 598 questionnaires were completed by the samples. 

### 2.4. Ethical Consideration

The Social Determinants of Health Research Center at Guilan University of Medical Sciences approved the study protocol under the code of IR.GUMS.REC.1394.52. Prior to the study, the permission to enter the study was obtained. The samples were informed of the study aim and process and were ensured of confidentiality of data. Those who were willing to take part in the study signed the informed consent form.

### 2.5. Data Analysis

Descriptive and inferential statistics were used via the SPSS v. 25 software (IBM, Armonk, NY, USA). Regarding the normal distribution of data, mean and standard deviation of social skill scores were calculated for cooperation, assertion, self-control, and the total social skills from the teachers’ and parents’ perspectives. To examine gender differences in social skill subscales in preschool and at home separately, the independent samples t-test was used based on the teacher’s (*α* < 0.05) and patent’s (α < 0.05) ratings. Given the categorization of the social skills level, the social skill subscales and total social skills of children were categorized into three categories (low, moderate, and high levels) for parents’ and teachers’ ratings separately. Afterwards, the Chi-square test was used to compare the percentages of social skill subscales between the teachers’ and parents’ ratings (α < 0.05). In addition, correlation analysis using the Pearson test was performed to examine the parent–teacher agreement ratings (α < 0.05). Moreover, the STATA software (Version 15, Stata Corporation, and College Station, TX, USA) was used to perform multivariable linear regression and investigate the association between sociodemographic variables (variables listed in Table 1) and social skill domains based on the parents’ and teachers’ ratings (α < 0.05). 

## 3. Results

Due to missing data (n = 17) and noncompliance with exclusion criteria (n = 35), 52 questionnaires were excluded from the data analysis and reporting. According to Table 1, the sociodemographic characteristics of the participants were categorized into three groups of child-related, child familial-related, and teacher- and preschool center-related variables. Most children were boys (57.9%), single children (52.2%), and the first child in the family (65.6%). Moreover, some children (45.6%) had a history of going to the kindergarten. Furthermore, 57.3% of the children’s mothers and fathers were 30–40 years old. The mothers (46.1%) had a high school education level, but the fathers (38.7%) had an academic education level. In addition, 74.4% of the mothers and 60% of the fathers were housekeepers and self-employed, respectively. Furthermore, the majority of the children (49.6%) had families with an income of $150–300 per month. The teachers were 30–40 years old (71.6%), had a bachelor education level (89.4%), and had above 10 years of teaching experience (46.7%). The children were studying at classes with 11–20 children in each classroom (42.7%), in single-sex classes (57.5%), in preschool centers under the supervision of the Ministry of Education region 1 (37%), and public schools (57.5%).

The social skills of the preschool children at home and in preschool based on gender differences and the teachers’ and parents’ ratings are shown in Table 2. From the teachers’ perspectives, boys achieved higher scores in the assertion domain than girls (*p* = 0.02) in preschool. However, from the parents’ perspectives, girls had higher scores in cooperation (*p* = 0.03), self-control (*p* = 0.001), and total social skill domains (*p* = 0.01) than boys at home. 

Table 3 shows the number and percentage of social skills in preschool children in three levels of low, moderate, and high from the teachers’ ratings in preschool and the parents’ ratings at home. From both perspectives, preschool children had a moderate level of all subscales of social skills and total social skills. The percentage of total social skills of children was 67.4% and 67.6% in the teachers’ and parents’ ratings, respectively. Further, differences between the teachers’ and parents’ ratings in the percentage of children in three categories were not statistically significant except for the cooperation domain (χ^2^ = 6.48 df = 2, n = 546, *p* = 0.03).

Table 4 presents the correlations between parents’–teachers’ agreement ratings of social skills in preschool children. A statistically significant correlation was reported between both cooperation ratings (r = 0.14, *p* < 0.001), the parents’ cooperation ratings, and the teachers’ total social skills ratings (r = 0.12, *p* = 0.003). In addition, a statistically significant correlation was found between both assertion ratings (r = 0.15, *p* < 0.001), the parents’ assertion ratings, and the teachers’ total social skills ratings (r = 0.10, *p* = 0.013). Additionally, there was a statistically significant correlation between the parents’ self-control ratings and the teachers’ cooperation ratings (r = 0.08, *p* = 0.03). Finally, in the total social skill domains, statistically significant correlations were found between the parents’ total social skills ratings and the teachers’ cooperation (r = 0.11, *p* = 0.005), assertion (r = 0.11, *p* = 0.009), and total social skills ratings (r = 0.13, *p* = 0.002).

The multivariable linear regression analysis to investigate the association between sociodemographic variables and social skill domain scores from the parents’ and teachers’ ratings is shown in Table 5. Only variables presented in this table showed statistically significant associations with at least one domain of social skills from the parents’ or the teachers’ perspectives. An inverse significant association was found between boys’ self-control (b = −0.46, 95% CI = −0.76, −0.16) and boys’ total social skills (b = −1.91, 95% CI = −3.66, −0.15), and child gender from the parents’ ratings. However, no statistically significant association was reported between the child’s gender and the teachers’ social skills ratings. In addition, an inverse association was found between self-control and the second birth rank (b = −0.43, 95% CI = −0.83, −0.03) and the third or more birth rank (b = −0.97, 95% CI = −1.93, −0.02) from the teachers’ ratings. Further, there were direct significant associations between the assertion domain (b = 2.08, 95% CI = 0.58, 3.58) and total social skills (b = 3.48, 95% CI = 0.65, 6.32), and 30–40 years of the fathers’ age and assertion domain (b = 2.21, 95% CI = 0.36, 4.06), and total social skills (b = 3.85, 95% CI = 0.35, 7.36) with the father’s age (>40 years) from the parents’ perspectives. From the teachers’ perspectives, an inverse relationship was reported between the cooperation domain and the father’s unemployment (b = −4.61, 95% CI = −9.11, −0.10). Additionally, an inverse association was found between children’s cooperation and teacher’s age >40 years (b = −2.43, 95% CI = −4.83, −0.03) from the parents’ ratings and teacher’s age 30–40 years (b = −2.01, 95% CI = −3.86, −0.17) from the teachers’ ratings. In addition, a direct relationship was found between children’s self-control and teachers with a master degree education level (b = 1.08, 95% CI = 0.03, 2.14) from the parents’ perspectives. Furthermore, direct relationships were found between children’s cooperation (b = 8.25, 95% CI = 12.13, 4.38), assertion (b = 3.14, 95% CI = 5.33, 0.96), self-control (b = 1.98, 95% CI = 2.88, 1.08), and total social skills (b = 13.38, 95% CI = 19.33, 7.43), and teachers with a master education level from the teachers’ perspectives. Furthermore, there were direct associations between children’s cooperation, assertion, self-control, total social skills, and teachers with 5–10 years of teaching experiences, and teachers with >10 years of teaching experiences based on the teachers’ perspectives. Moreover, there were inverse associations between children’s cooperation (b = −3.42, 95% CI = −6.39, −0.47), assertion (b = −2.02, 95% CI = −3.69, −0.36), self-control (b = −0.78, 95% CI = −1.47, −0.10), and total social skills (b = −6.23, 95% CI = −10.76, −1.71) and >30 children in each preschool classroom from the teachers’ ratings. Finally, a direct relationship was found between children’s assertion and coeducational classes from the parents’ perspectives (b = 2.08, 95% CI = 0.095, 4.07).

## 4. Discussion

The findings of this study revealed that the social skills of girls were more than those of boys at home. The majority of children had a moderate level of social skills from the parents’ and teachers’ perspectives. A modest parent–teacher agreement was found in most domains of social skills. Further, statistically significant associations were reported between children’s social skill domains and the child’s birth rank, father’s age, father’s job, teacher’s age, teacher’s education level, teacher’s teaching experience, and preschool classroom in terms of the numbers of children and the type of classroom. 

From the teachers’ ratings, no correlation was found between both genders except high assertion scores in boys in preschool. From the parents’ ratings, girls had higher social skills in the subscale of cooperation, self-control, and total social skills at home. The reports of studies in Western societies showed that girls had more social skills than boys [5,15,40,41]. However, previous studies on Iranian preschool children showed no significant relationship between total social skills and the gender of children from teachers’ perspectives [29,30]. Taverna et al. showed that cultural differences had significant roles in the creation of different expectations in children [31]. Further, cultural stereotypes and reactions influence gender roles and gender-related behaviors [22]. In Eastern countries, female children are expected to be chaste, modest, and gentle in community settings than boys during childhood and other periods of their life. In this regard, Iranian female children often display their capabilities less, especially social skills to others in social environments [27,32,42]. In addition, due to social expectations, Iranian teachers show less tolerance to social problems in female children than male children and as a result, they evaluate the social skills of girls lower than boys [29]. 

In addition to cultural characteristics that encourage Iranian girls to become cooperative, submissive to tasks, kind, responsive, and empathic at home [32,42], based on the Bandura’s social cognitive learning theory, children are more likely to prefer the same behavioral patterns of their homogenous parents. Therefore, girls are adapted to social behaviors in preschool, such as cooperation and accountability, but boys’ modeling of behaviors is more active and aggressive [43,44]. Further, educational settings prescribe gender roles and ask children to demonstrate related social skills in a variety of environments. Men are widely portrayed in an active manner, but females’ presence is mainly limited to traditional roles at home [45]. Therefore, in this study, girls’ social skills at home were evaluated to be higher in cooperation, self-control, and total social skills than those of boys at home, which was consistent with the results of other studies conducted in Iran [22,30]. Higher assertion in boys can be attributed to the abovementioned reasons [43,44,45,46].

In addition to gender differences in social skills described in this study, gender differences in other areas of development such as self-concept and environmental empathy are present [47,48]. Self-concept as a multifaceted belief system is evaluated in relation to the environment. According to Muthuri and Arasa’s study, males had a higher overall self-concept than females [49]. Environmental empathy is the individual’s ability to understand and respond to the environment. Musitu-Ferrer et al. reported that females showed higher environmental emotional empathy than males [47].

The results of present study showed that the majority of children had a moderate level of cooperation, assertion, self-control, and total social skills from both the teachers’ and parents’ perspectives. In the preschool age, children get the first experiences of socialization at home and school. Therefore, their initial experiences are reported at a moderate level [14,29,30,50].

There was a significant parent–teacher agreement on social skills in all domains except self-control, but it was modest in magnitude. The social competence is multifaceted, and children’s social behaviors are different at home and school. Therefore, children’s behaviors vary across situations [51]. This finding is supported by the teacher–parent agreement on children’s behaviors [14,52,53]. Significant correlations between the parents’ and teachers’ ratings support the notion of cross-situation consistency in children’s social behaviors [14].

The findings of this study showed an inverse significant association between the birth rank and self-control from the teachers’ ratings. In general, higher birth ranks have an inverse effect on cognitive and social development [54]. Self-control is an individual’s ability to control ambitions to achieve long-term goals [55]. Parents are worried for their firstborn children, which affects how they bring them up. Therefore, firstborn children achieve more self-control than other children [56,57].

In this study, the social skills of children with older fathers were found to be greater than those of children with younger fathers. Parenting as a complex activity consists of special methods and behaviors that interact with each other and influence the child’s social development. Therefore, parenting skills are affected by cultural, economic conditions, and the parent’s knowledge [58]. The authoritative style of parenting is most common among intact European American families and helps children to gain higher levels of social competence [59]. Yousefi’s study in Iran showed that the authoritative parental style was associated with low social skills in children [60]. Accordingly, in the Iranian culture, permissive parenting by parents aged 35–45 years leads to higher development of social skills in children [59,60]. With the increase of parents’ age, they become less negligent [61]. Furthermore, an association between increasing parental age and parental knowledge is available. Therefore, higher parental knowledge leads to a higher quality of the home environment and less possibility of children’s neglect or abuse [62]. Parents with greater knowledge provide verbal and physical stimulations to children, utilize less negative disciplinary strategies, and interact more with children [63]. In addition, economic conditions, the childhood environment, and the family’s standard of living are improved with parents’ age [64,65].

Children with teachers that have a higher education level had higher social skills. Education in early childhood is important for social skill development in future life [66]. Experienced teachers engage themselves in sensitive and stimulating interactions with children at early childhood and create richer learning experiences [67]. Further, children educated by qualified early childhood teachers are more sociable and have higher levels of language skills and cognitive abilities [68]. 

In this study, children educated by more experienced teachers achieved higher scores in social skills. It can be said that teachers with more work experience and those with more in-service training can improve children’s life skills [69,70]. Such work experience is needed for better time management, evaluation methods, practices and feedback given on children’s ability, and social performance [71]. Such teachers educate children on how to solve social problems and adapt to negative life situations [19,72]. 

In this study, children studying in classrooms with more than 30 children had lower social skills. There is an inverse relationship between classroom size and social learning, so the learning of social skills in large classrooms can be worse than that in small classrooms [73]. A large classroom size reduces the frequency and quality of interactions and feedback between teacher and children [74]. Small classrooms create individualized instructions, higher-quality instructions, increased teacher morale, fewer disruptions, less child misbehavior, and greater engagement of children in social activities [75].

This study revealed that children in coeducational classes had more social skills in all domains except self-control, but this was only significant in the assertion domain. Studies reported the positive effects of single-sex education on children’s achievement compared to coeducational education [76,77,78]. By contrast, the major benefits of coeducation could be personal and social development in children. Actually, educating both genders creates more real life experiences and makes it easier to practice working in a mixed environment [78]. In addition, it prevents the development of mixed-gender anxiety and avoidance of mixed-gender situations [79]. Coeducation can better prepare young children for cross-gender interactions and integration into society [77].

### 4.1. Limitations

Generalizability of findings to preschool children in other contexts needs further studies due to cultural differences affecting the development of social skills in children. The study sample size was limited to children at the 6th year of age and living in an urban area, but no data were collected from children, which should be considered in future studies. 

### 4.2. Contributions and Implications

Data regarding children social skills were collected from the parents’ and teachers’ perspectives as reliable sources of information about children’s development. Therefore, our findings can improve the international knowledge of social skills in children and gender differences affecting children’s adjustment. In addition, this study identified the importance of a child’s birth rank, father’s age, father’s job, teacher’s age, teacher’s education, teacher’s experience, and preschool classrooms influencing social skills. Policymakers can use our findings to improve the development of children’s social skills [5,66]. More attention should be given to younger parents in term of parental tasks and to enhance their positive parental behaviors, which can lead to better children’s social development [80].

## 5. Conclusions

Girls had higher cooperation, self-control, and total social skills at home from the parents’ perspectives, but the teachers’ perspectives showed no difference in social skill domains between genders except for a higher level of assertion in boys in preschool. The majority of children had moderate social skills in all domains from the parents’ and teachers’ ratings, and no difference between them was reported, except in cooperation. In addition, a modest parent–teacher agreement in all domains of social skills except self-control was found. Furthermore, statistically significant associations were reported between the parents’ and teachers’ ratings of children’s social skills, and the child’s birth rank, father’s age, father’s job, teacher’s age, teacher’s education, teacher’s experience, preschool classroom in terms of the numbers of children, and the type of classroom. Accordingly, the risk of problems with social skills was reported to be relatively low. More attention should be given to the family status, teacher’s, and preschool center’s characteristics to improve children’s social skills. 

## Figures and Tables

**Table 1:** The participants’ characteristics according to demographic and social features.

Variables		Number (%)
**Child-Related Variables**		
Child’s gender	Girl	230 (42.1)
	Boy	316 (57.9)
Child’s birth rank	First	358 (65.6)
	Second	166 (30.4)
	Third or more	22 (4)
Number of children in the family	One	285 (52.2)
	Two	223 (40.8)
	Third or more	38 (7)
Going to the kindergarten	Yes	249 (45.6)
	No	297 (54.4)
**Child Familial-Related Variables**		
Mother’s age	<30	173 (31.7)
	30–40	313 (57.3)
	>40	60 (11)
Father’s age	<30	56 (10.3)
	30–40	313 (57.3)
	>40	177 (32.4)
Mother’s education level	Elementary	21 (3.8)
	Guidance school	65 (11.9)
	High school	251 (46.1)
	Academic	208 (38.2)
Father’s education level	Elementary	40 (7.3)
	Guidance school	102 (18.7)
	High school	193 (35.3)
	Academic	211 (38.7)
Mother’s job	Employed	140 (25.6)
	Housekeeper	406 (74.4)
Father’s job	Employee	210 (38.5)
	Self-employed	328 (60)
	Unemployed	8 (1.5)
Family’s income (USA dollar)	<150	67 (12.3)
	150–300	271 (49.6)
	>300	208 (38.1)
**Teachers/and Preschool Centers/Related Variables**		
Teacher age (year)	<30	76 (13.9)
	30–40	391 (71.6)
	>40	79 (14.5)
Teacher’s education level	Associate	36 (6.6)
	Bachelor	488 (89.4)
	Master	22 (4)
Teacher’s teaching experience (year)	<5	123 (22.5)
	5–10	168 (30.8)
	>10	255 (46.7)
Number of children in each classroom	≤10	31 (5.7)
	11–20	233 (42.7)
	21–30	122 (22.3)
	>30	160 (29.3)
Type of classroom	Single-sex	314 (57.5)
	Coeducational	232 (42.5)
Type of organizational affiliation	Ministry of Education 1	202 (37)
	Ministry of Education 2	181 (33.1)
	Welfare Organization	163 (29.9)
Preschool type	Public	314 (57.5)
	Nonprofit	69 (12.6)
	Private	163 (29.9)

**Table 2:** Gender differences in social skills from the teachers’ and parents’ perspectives.

Social Skill Domains	Teacher’s Rating				
	Girls Mean (SD)	Boys Mean (SD)	Total Mean (SD)	*Df*	*p*-Value *
Cooperation	25.70 (5.76)	25.24 (6.52)	25.44 (6.21)	544	0.39
Assertion	9.63 (3.93)	10.30 (3.13)	10.20 (3.50)	544	0.02
Self-control	3.94 (1.45)	3.76 (1.39)	3.84 (1.42)	544	0.15
Total social skills	39.13 (9.60)	39.43 (9.61)	39.30 (9.60)	544	0.96
	**Parent’s Rating**				
Cooperation	26.58 (5.44)	25.61 (5.12)	26.02 (5.27)	544	0.03
Assertion	22.86 (4.97)	22.23 (5.04)	22.49 (5.01)	544	0.152
Self-control	5.07 (1.62)	4.69 (1.58)	4.85 (1.60)	544	0.001
Total social skills	54.51 (9.77)	52.54 (9.35)	53.37 (9.57)	544	0.01

* Independent samples t-test; *Df*: degree of freedom.

**Table 3:** The social skills of preschool children from the teachers’ and parents’ perspectives and related differences (n = 546).

Social Skill Domains	Teacher’s Rating n (%)			Parent’s Rating n (%)			Chi-Square *	
	Low	Moderate	High	Low	Moderate	High	Value	*p*-Value
Cooperation	93 (17)	355 (65)	98 (18)	73 (13.4)	394 (72.1)	79 (14.5)	6.48	0.03
Assertion	76 (14)	367 (67.2)	103 (18.8)	99 (18.1)	359 (65.8)	88 (16.1)	4.28	0.11
Self-control	93 (17)	395 (72.3)	58 (10.7)	98 (17.9)	368 (67.4)	80 (14.7)	4.59	0.10
Total social skills	84 (15.4)	368 (67.4)	94 (17.2)	85 (15.6)	369 (67.6)	92 (16.8)	0.02	0.98

* Chi-square test.

**Table 4:** Correlation coefficients of the parents’ and the teachers’ ratings (n = 546).

SSRS–Parent	SSRS–Teacher			
	Cooperation	Assertion	Self-Control	Total SSRS
Cooperation	0.14 ***	0.07 ^-^	0.06	0.12 **
Assertion	0.04	0.15 ***	0.01	0.10 *
Self-control	0.08 *	0.03	0.07 ^-^	0.08 ^-^
Total SSRS	0.11 **	0.11 **	0.05	0.13 **

^-^*p* < 0.10; * *p* < 0.05; ** *p* < 0.01; *** *p* < 0.001.

**Table 5:** Association between sociodemographic variables and social skill domains from the parents’ and teachers’ ratings.

Variable	SSRS–Parent				SSRS–Teacher			
	C	A	S	T	C	A	S	T
	b *	*b*	*b*	*b*	*b*	*b*	*b*	*b*
Child’s gender								
Girl	ref	ref	ref	ref	ref	ref	ref	ref
Boy	−0.76	−0.68	−0.46 **	−1.91 *	−0.22	0.38	−0.12	0.03
Child’s birth rank								
First	ref	ref	ref	ref	ref	ref	ref	ref
Second	0.21	0.75	0.16	1.14	−0.54	0.79	−0.43 *	−0.18
Third or more	0.20	0.64	0.28	1.13	−0.01	0.53	−0.97 *	−0.45
Father’s age (years)								
<30	ref	ref	ref	ref	ref	ref	ref	ref
30–40	1.36	2.08 **	0.08	3.48 *	0.26	0.44	−0.00	0.70
<40	1.50	2.21 **	0.13	3.85 *	0.81	0.35	0.05	1.23
Father’s job								
Employee	ref	ref	ref	ref	ref	ref	ref	ref
Self-employed	−0.88	−0.24	−0.09	−1.22	0.00	0.17	0.21	0.40
Unemployed	−1.00	−2.11	−0.35	−3.47	−4.61 *	−0.41	−0.19	−5.21
Teacher age (years)								
<30	ref	ref	ref	ref	ref	ref	ref	ref
30–40	−0.64	−0.99	0.04	−1.60	−2.01 *	−0.04	−0.02	−2.02
>40	−2.43 *	−2.01	0.21	−4.23	−0.39	0.01	−0.02	−0.40
Teacher education								
Associate	ref	ref	ref	ref	ref	ref	ref	ref
Bachelor	2.15	0.01	0.02	2.11	1.51	0.40	0.49	1.60
Master	2.91	1.11	1.08 *	2.94	8.25 ***	3.14 **	1.98 ***	13.38 ***
Teacher teaching experience (years)								
<5	ref	ref	ref	ref	ref	ref	ref	ref
5–10	−0.43	−0.34	−0.30	−1.08	4.31 ***	1.14 *	0.58 **	6.04 ***
>10	0.24	0.81	−0.23	0.82	4.59 ***	1.89 ***	0.98 ***	7.46 ***
Number of children in each classroom								
≤10	ref	ref	ref	ref	ref	ref	ref	ref
11–20	0.07	0.67	0.13	0.89	−0.70	0.24	−0.17	−0.64
21–30	−0.36	0.59	−0.25	−0.01	−0.53	0.16	−0.13	−0.50
>30	0.20	0.68	−0.03	0.84	−3.42 *	−2.02 *	−0.78 *	−6.23 **
Type of classroom								
Single-sex	ref	ref	ref	ref	ref	ref	ref	ref
Coeducational	0.39	2.08 *	−0.44	2.07	0.99	1.00	−0.34	1.65

* *b* coefficient was obtained according to the multivariable linear regression A: Assertion, C: Cooperation, S: Self-control, T: Total social skills; * *p* < 0.05, ** *p* < 0.01, *** *p* < 0.001.

## References

[1] Salmani-Barough N., Sharifi-Neiestanak N., Kazemnejad A., Pashaeypoor S. (2003). Self-concept and influential factors on it in the street children aged 6–12 years. J. Hayat.

[2] Hockenberry M.J., Wilson D. (2013). Wong’s Essentials of Pediatric Nursing.

[3] Teodoro M.L.M., Kappler K.C., de Lima Rodrigues J., de Freitas P.M., Haase V.G. (2005). The Matson Evaluation of Social Skills with Youngsters (MESSY) and its adaptation for Brazilian children and adolescents. Interam. J. Psychol..

[4] Takahashi Y., Okada K., Hoshino T., Anme T. (2015). Developmental trajectories of social skills during early childhood and links to parenting practices in a Japanese sample. PLoS ONE.

[5] Pecjak S., Puklek Levpuscek M., Valencic Zuljan M., Kalin J., Peklaj C. (2009). Students’ social behaviour in relation to their academic achievement in primary and secondary school: Teacher’s perspective. Psihol. Teme.

[6] Aksoy P., Baran G. (2010). Review of studies aimed at bringing social skills for children in preschool period. Procedia-Soc. Behav. Sci..

[7] Gulay H., Akman B. (2009). Social Skills in Preschool Period.

[8] Kramer T.J., Caldarella P., Christensen L., Shatzer R.H. (2010). Social and emotional learning in the kindergarten classroom: Evaluation of the strong start curriculum. Early Child. Educ. J..

[9] Ziv Y. (2013). Social information processing patterns, social skills, and school readiness in preschool children. J. Exp. Child Psychol..

[10] Lodder G., Goossens L., Scholte R., Engels R., Verhagen M. (2016). Adolescent loneliness and social skills: Agreement and discrepancies between self-, meta-, and peer-evaluations. J. Youth Adolesc..

[11] Powless D.L., Elliott S.N. (1993). Assessment of social skills of Native American preschoolers: Teachers’ and parents’ ratings. J. Sch. Psychol..

[12] Whitted K.S. (2011). Understanding how social and emotional skill deficits contribute to school failure. Prev. School Fail. Altern. Educ. Child. Youth.

[13] Gresham F.M., Elliott S.N. (1990). Social Skills Rating System: Preschool, Elementary Level.

[14] Tan T.X., Camras L.A. (2011). Social skills of adopted Chinese girls at home and in school: Parent and teacher ratings. Child. Youth Serv. Rev..

[15] Olcer S., Aytar A.G. (2014). A comparative study into social skills of five-six year old children and parental behaviors. Procedia-Soc. Behav. Sci..

[16] Cimen N., Kocyigit S. (2010). A study on the achievement level of social skills objectives and outcomes in the preschool curriculum for six-year-olds. Procedia-Soc. Behav. Sci..

[17] Dobbins N., Higgins K., Pierce T., Tandy R.D., Tincani M. (2010). An analysis of social skills instruction provided in teacher education and in-service training programs for general and special educators. Remedial Spec. Educ..

[18] Lynne Lane K., Stanton-Chapman T., Roorbach Jamison K., Phillips A. (2007). Teacher and parent expectations of preschoolers’ behavior: Social skills necessary for success. Top. Early Child. Spec. Educ..

[19] Zhang X., Nurmi J.E. (2012). Teacher–child relationships and social competence: A two-year longitudinal study of Chinese preschoolers. J. Appl. Dev. Psychol..

[20] Myers S.S., Pianta R.C. (2008). Developmental commentary: Individual and contextual influences on student–teacher relationships and children’s early problem behaviors. J. Clin. Child Adolesc. Psychol..

[21] Domitrovich C.E., Cortes R.C., Greenberg M.T. (2007). Improving young children’s social and emotional competence: A randomized trial of the preschool “PATHS” curriculum. J. Prim. Prev..

[22] Abdi B. (2010). Gender differences in social skills, problem behaviours and academic competence of Iranian kindergarten children based on their parent and teacher ratings. Procedia-Soc. Behav. Sci..

[23] Yoleri S. (2017). Teacher-child relationships in preschool period: The roles of child temperament and language skills. Int. Electron. J. Elem. Educ..

[24] Koch H., Kastner-Koller U., Deimann P., Kossmeier C., Koitz C., Steiner M. (2011). The development of kindergarten children as evaluated by their kindergarten teachers and mothers. Psychol. Test Assess. Model..

[25] Veenstra R., Lindenberg S., Oldehinkel A.J., De Winter A.F., Verhulst F.C., Ormel J. (2008). Prosocial and antisocial behavior in preadolescence: Teachers’ and parents’ perceptions of the behavior of girls and boys. Int. J. Behav. Dev..

[26] Maleki M., Chehrzad M.M., Kazemnezhad Leyli E., Mardani A., Vaismoradi M. (2019). Social Skills in Preschool Children from Teachers’ Perspectives. Children.

[27] Mohamed A.H. (2018). Gender as a moderator of the association between teacher-child relationship and social skills in preschool. Early Child Dev. Care.

[28] Tan K., Oe J.S., Hoang Le M.D. (2018). How does gender relate to social skills? Exploring differences in social skills mindsets, academics, and behaviors among high-school freshmen students. Psychol. Sch..

[29] Shahim S. (2005). Standardization of Social Skills Rating System for Preschool Children. Iran. J. Psychiatry Clin. Psychol..

[30] Sheikhzakaryaie N., Nikpour S., Ameri Z., Haghani H. (2012). Gender differences in social skills of Iranian preschool children. Arch. Sci..

[31] Taverna L., Bornstein M.H., Putnick D.L., Axia G. (2011). Adaptive behaviors in young children: A unique cultural comparison in Italy. J. Cross-Cult. Psychol..

[32] Nourani K. (1999). Social Skills and Adaptive Behavior of Iranian Preschoolers, Teachers’ and Parents’ Ratings. Ph.D. Thesis.

[33] Dougherty L.R., Leppert K.A., Merwin S.M., Smith V.C., Bufferd S.J., Kushner M.R. (2015). Advances and directions in preschool mental health research. Child Dev. Perspect..

[34] Arani A.M., Kakia M.L., Karimi M.V. Assessment in Education in Iran. http://www.nwu.ac.za/sites/www.nwu.ac.za/files/files/p-saeduc/New_Folder_1/3_Assessment%20in%20education%20in%20Iran.pdf.

[35] Van Horn M.L., Atkins-Burnett S., Karlin E., Ramey S.L., Snyder S. (2007). Parent ratings of children’s social skills: Longitudinal psychometric analyses of the Social Skills Rating System. Sch. Psychol. Q..

[36] Shahim S. (2004). Reliability of the social skills rating system for preschool children in Iran. Psychol. Rep..

[37] Hess M., Scheithauer H., Kleiber D., Wille N., Erhart M., Ravens-Sieberer U. (2014). The parent version of the preschool Social Skills Rating System: Psychometric analysis and adaptation with a German preschool sample. J. Psychoeduc. Assess..

[38] Manz P.H., Fantuzzo J.W., McDermott P.A. (1999). The parent version of the preschool social skills rating scale: An analysis of its use with low-income, ethnic minority children. Sch. Psychol. Rev..

[39] Maleki M., Mitra Chehrzad M., Reza Masouleh S., Kazemnezhad Leyli E. (2018). Social Skills in Preschool Children From Their Parents’ Points of View. J. Holist. Nurs. Midwifery.

[40] Gomes R.M.S., Pereira A.S. (2014). Influence of age and gender in acquiring social skills in Portuguese preschool education. Psychology.

[41] Anme T., Shinohara R., Sugisawa Y., Tong L., Tanaka E., Watanabe T., Onda Y., Kawashima Y., Hirano M., Tomisaki E. (2010). Gender differences of children’s social skills and parenting using Interaction Rating Scale (IRS). Procedia-Soc. Behav. Sci..

[42] Daniel E.L. (2006). Culture and Customs of Iran.

[43] Abdi B. (2008). Social skills and behavior problems of iranian preschoolers. Sci. J. Manag. Syst..

[44] Bussey K., Bandura A. (1999). Social cognitive theory of gender development and differentiation. Psychol. Rev..

[45] Foroutan Y. (2019). Formation of gender identity in the Islamic republic of Iran: Does educational institution matter?. J. Beliefs Values.

[46] Hollandsworth J.G., Wall K.E. (1977). Sex differences in assertive behavior: An empirical investigation. J. Couns. Psychol..

[47] Musitu-Ferrer D., Esteban Ibáñez M., León C., Garcia O. (2019). Is school adjustment related to environmental empathy and connectedness to nature?. Psychosoc. Interv..

[48] Garcia F., Martínez I., Balluerka N., Cruise E., Garcia O.F., Serra E. (2018). Validation of the Five-Factor Self-Concept Questionnaire AF5 in Brazil: Testing factor structure and measurement invariance across language (Brazilian and Spanish), gender and age. Front. Psychol..

[49] Muthuri R., Arasa J. (2017). Gender Differences in Self-Concept Among a Sample of Students of the United States; International University in Africa. Ann. Behav. Sci..

[50] Garmaroudi G., Vahdaninia M. (2006). Social health; assessment of students’ social skills. Payesh.

[51] Junttila N., Voeten M., Kaukiainen A., Vauras M. (2006). Multisource assessment of children’s social competence. Educ. Psychol. Meas..

[52] Dinnebeil L.A., Sawyer B.E., Logan J., Dynia J.M., Cancio E., Justice L.M. (2013). Influences on the congruence between parents’ and teachers’ ratings of young children’s social skills and problem behaviors. Early Child. Res. Q..

[53] Merrell K.W., Popinga M.R. (1994). Parent-teacher concordance and gender differences in behavioral ratings of social skills and social-emotional problems of primary-age children with disabilities. Diagnostique.

[54] De Haan M., Plug E., Rosero J. (2014). Birth order and human capital development evidence from ecuador. J. Hum. Resour..

[55] Pan Q., Zhu Q. (2018). Development of self-control in early childhood—A growth mixture modeling approach. Cogent Psychol..

[56] Eisenman R. (1992). Birth order, development and personality. Acta Paedopsychiatr. Int. J. Child Adolesc. Psychiatry.

[57] Nouhjah S., Mokhveli Khazaei F. (2014). Assessment of motor development of children attending health centers of Dezful City using World Health Organization standard indexes. J. Paramed. Sci. Rehabil..

[58] Latifi Z., Moradi F. (2016). Parenting practices in Iran. Int. J. Humanit. Cult. Stud..

[59] Kazemi A., Ardabili H.E., Solokian S. (2010). The association between social competence in adolescents and mothers’ parenting style: A cross sectional study on Iranian girls. Child Adolesc. Soc. Work J..

[60] Yousefi F. (2007). The relationships between parenting styles, social skills, and some aspects of self-concept among high school students. Daneshvar Raftar.

[61] Kashahu L., Dibra G., Osmanaga F., Bushati J. (2014). The relationship between parental demographics, parenting styles and student academic achievement. Eur. Sci. J..

[62] Morawska A., Winter L., Sanders M. (2009). Parenting knowledge and its role in the prediction of dysfunctional parenting and disruptive child behaviour. Child Care Health Dev..

[63] Huang K.Y., Caughy M.O.B., Genevro J.L., Miller T.L. (2005). Maternal knowledge of child development and quality of parenting among White, African-American and Hispanic mothers. J. Appl. Dev. Psychol..

[64] Carslake D., Tynelius P., Van Den Berg G., Smith G.D., Rasmussen F. (2017). Associations of parental age with health and social factors in adult offspring. Methodological pitfalls and possibilities. Sci. Rep..

[65] Conger R.D., Conger K.J., Martin M.J. (2010). Socioeconomic status, family processes, and individual development. J. Marriage Fam..

[66] Mischo C., Wahl S., Strohmer J., Wolf C. (2013). Does early childhood teacher education affect students’ cognitive orientations?. J. Educ. Train. Stud..

[67] Fukkink R., Jilink L., den Kelder R.O., Zeijlmans K., Bollen I., Koopman L. (2019). The development of interaction skills in preservice teacher education: A mixed-methods study of Dutch pre-service teachers. Early Child. Educ. J..

[68] Lobman C., Ryan S., McLaughlin J. (2005). Reconstructing Teacher Education to Prepare Qualified Preschool Teachers: Lessons from New Jersey. Early Child. Res. Pract..

[69] Arefi M., Fathi V.K., Naderi R. (2009). Academic and professional knowledge of primary school teachers about learning theories: Evidence from primary school teachers of Hamedan. Educ. Innov..

[70] Balta N., Arslan M., Duru H. (2015). The effect of in-service training courses on teacher achievement: A meta-analysis study. J. Educ. Train. Stud..

[71] Peter M. (2012). Influence of Teacher Training on the Performance of Students in Mixed Secondary School in Gem District, Kenya.

[72] O’Connor E.E., Dearing E., Collins B.A. (2011). Teacher-child relationship and behavior problem trajectories in elementary school. Am. Educ. Res. J..

[73] Karakaya F., Ainscough T.L., Chopoorian J. (2001). The effects of class size and learning style on student performance in a multimedia-based marketing course. J. Mark. Educ..

[74] Cuseo J. (2007). The empirical case against large class size: Adverse effects on the teaching, learning, and retention of first-year students. J. Fac. Dev..

[75] Hattie J. (2005). The paradox of reducing class size and improving learning outcomes. Int. J. Educ. Res..

[76] Woodward L.J., Fergusson D.M., Horwood L.J. (1999). Effects of single-sex and coeducational secondary schooling on children’s academic achievement. Aust. J. Educ..

[77] Mael F.A. (1998). Single-sex and coeducational schooling: Relationships to socioemotional and academic development. Rev. Educ. Res..

[78] Malik R.A. (2013). Differential Effects of Single Sex versus Coed Education on the Personality Development of Primary School Students. Pak. J. Soc. Sci..

[79] Wong W.I., Shi S.Y., Chen Z. (2018). Students from single-sex schools are more gender-salient and more anxious in mixed-gender situations: Results from high school and college samples. PLoS ONE.

[80] Zahra E.D., Nazanin V., Reza E.M., Sima K., Zohreh S. (2014). Implementation of mother-training program to improve parenting in pre-school age children: A randomized-controlled trial. N. Am. J. Med. Sci..

